# Potential harmful health effects of inhaling nicotine-free shisha-pen vapor: a chemical risk assessment of the main components propylene glycol and glycerol

**DOI:** 10.1186/s12971-015-0038-7

**Published:** 2015-06-27

**Authors:** Anne S Kienhuis, Lya G Soeteman-Hernandez, Peter MJ Bos, Hans WJM Cremers, Walther N Klerx, Reinskje Talhout

**Affiliations:** National Institute for Public Health and the Environment (RIVM), P.O. Box 1, 3720 BA Bilthoven, The Netherlands

**Keywords:** Shisha-pen, E-cigarettes, Chemical risk assessment, Propylene glycol, Glycerol

## Abstract

**Background:**

A shisha-pen is an electronic cigarette variant that is advertised to mimic the taste of a water pipe, or shisha. The aim of this study was to assess the potential harmful health effects caused by inhaling the vapor of a nicotine-free shisha-pen.

**Methods:**

Gas chromatography analysis was performed to determine the major components in shisha-pen vapor. Risk assessment was performed using puff volumes of e-cigarettes and “normal” cigarettes and a 1-puff scenario (one-time exposure). The concentrations that reached the airways and lungs after using a shisha-pen were calculated and compared to data from published toxicity studies.

**Results:**

The main components in shisha-pen vapor are propylene glycol and glycerol (54%/46%). One puff (50 to 70 mL) results in exposure of propylene glycol and glycerol of 430 to 603 mg/m^3^ and 348 to 495 mg/m^3^, respectively. These exposure concentrations were higher than the points of departure for airway irritation based on a human study (propylene glycol, mean concentration of 309 mg/m^3^) and a rat study (glycerol, no-observed adverse effect level of 165 mg/m^3^).

**Conclusions:**

Already after one puff of the shisha-pen, the concentrations of propylene glycol and glycerol are sufficiently high to potentially cause irritation of the airways. New products such as the shisha-pen should be detected and risks should be assessed to inform regulatory actions aimed at limiting potential harm that may be caused to consumers and protecting young people to take up smoking.

## Background

A shisha-pen is an electronic cigarette (e-cigarette) variant that is advertised to mimic the taste of a water pipe, or shisha. It is available with many flavors, such as strawberry, vanilla, and cola. The shisha-pen operates in the same manner as an e-cigarette, it can be disposable or rechargeable and refillable, and it is available with and without nicotine [1].

**Figure 1:**
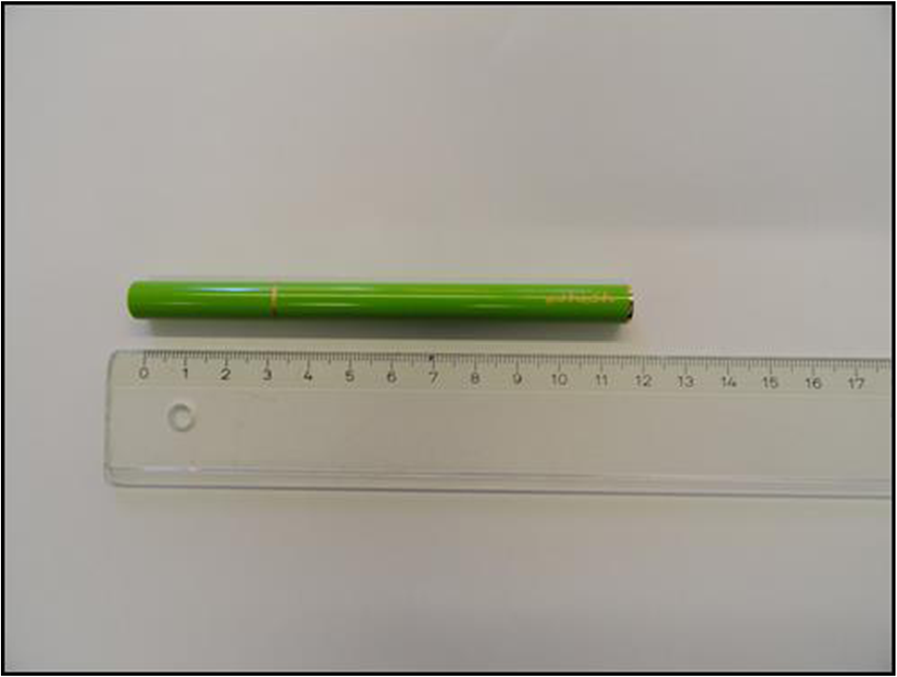
**Shisha-pen, apple flavor.**

**Figure 2:**
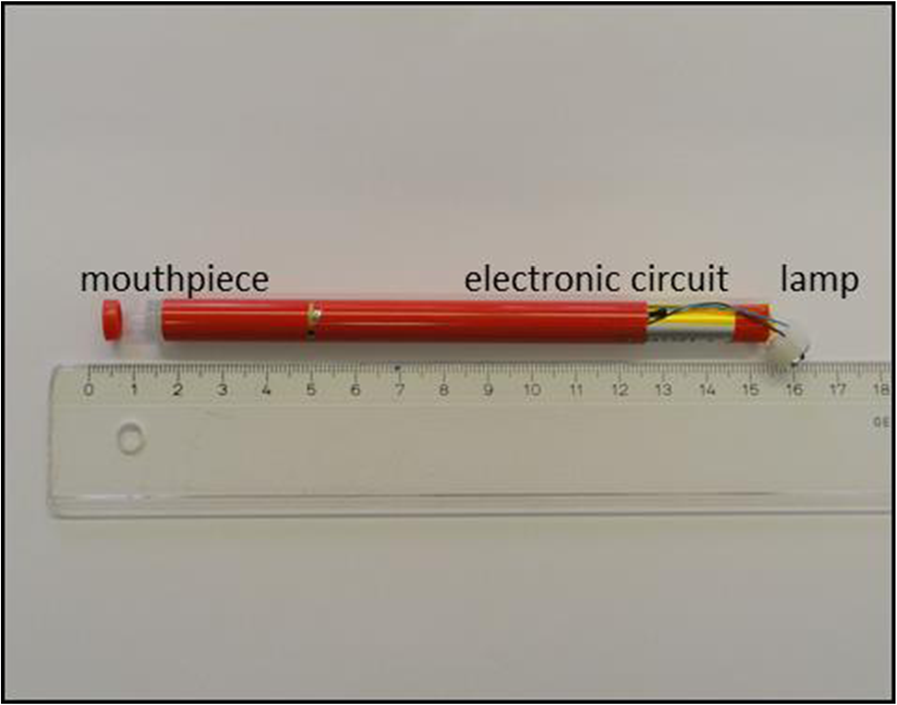
**Shisha-pen, dismantled, strawberry flavor.**

A shisha-pen is a pen that has a bulb in the shape of a diamond in one end and a mouthpiece with a small nozzle hole in the other end (Figure 1). The casing includes an electrical circuit with a battery and a coil that is coupled via a wire to a gauze pad soaked in liquid. When sucking on the mouthpiece, the electrical circuit is closed and the small wire connected to the gauze pad becomes warm, additionally the shisha-pen lamp is activated and lights up. As soon as the coil is heated, the liquid evaporates generating vapor which is inhaled. When air is no longer sucked in via the mouthpiece, the electronic circuit is interrupted and the wire cools down and the lamp turns off. The shisha-pen is ready for the next “air pull” via the mouthpiece until all the liquid in the gauze is evaporated. A dismantled shisha-pen is shown in Figure 2.

Shisha-pens can be purchased online and in stores where tobacco products are generally sold. In the Netherlands, there has been a media-hype about popularity of nicotine-free shisha-pens among elementary school children [2]. Concerns were raised whether these nicotine-free shisha-pens may act as a gateway product, facilitating later uptake of tobacco smoking among children and whether use of the shisha-pen, i.e. inhaling its contents, is actually safe. The purpose of the current study was to identify potential harmful health effects caused by exposure of consumers to the contents of the shisha-pen. To this aim, we assessed the chemical composition of the liquid and the vapor of the shisha-pen to estimate exposure. In addition, risk assessment on the major chemical components that emerge in the vapor of the shisha-pen was performed by selecting relevant toxicity studies and comparing them to exposure.

## Methods

Disposable, nicotine-free shisha-pens (3 strawberry, 1 apple and 1 grape) were bought in a local store. Constitution of the liquid and vapor of shisha-pens was analyzed using gas chromatography (GC) on a Varian GC 3900/FID. Both liquid (method 1) and vapor (method 2) were separated on a CP-WAX 52CB (25 m × 0.25 mm 1,2 μm) column. Galaxie software was used for quantification and identification of peaks. The analytical conditions employed were as follows: volume injected 1 μl, flow 2.5 mL/min, injector temperature 220°C, detector temperature 260°C, split ratio 1:50 and oven temperature from 160°C to 230°C with stepped temperature program: 3 minutes at 160°C, with 10°C per minute to 230°C, 10 minutes at 230°C. Calibration curves for propylene glycol and glycerol were linear, from 0.008 mg/mL (detection limit) to 4.0 mg/mL.

For method 1 (humectants in liquid), a shisha-pen (strawberry) was dismantled. All parts of the shisha-pen and their operation were described (see Results section). The gauze pad that contains the liquid was rinsed with 50 mL methanol. The proportion propylene glycol/glycerol was determined using the GC-FID method, using the settings as described above.

For method 2 (humectants in vapor), shisha-pens (2 strawberry, 1 apple, 1 grape) were smoked on a home-build one-channel smoking machine, using the ISO smoking regime (35 cm^3^ puff volume; 2 second puff duration; puff frequency of once per minute). Four to ten puffs (strawberry n = 10, strawberry n = 5, apple n = 5, grape n = 4) of 35 mL were captured on a Cambridge filter and extracted with 50 mL methanol. Propylene glycol and glycerol were determined using the GC-FID method, using the settings as described above.

Using the same GC-FID method, the presence of tri-ethyleneglycol, di-ethyleneglycol and nicotine was determined. Additionally, pyrolysis was performed on one strawberry shisha-pen at 140°C using a PTV injector and gas chromatography–mass spectrometry (GC-MS) iontrap varian 3800 with varian iontrap MS225 to determine the presence of components that would be expected in tobacco smoke.

Risk assessment was performed for the major components found in the shisha-pen vapor, according to a procedure recently developed for smoking of cigarettes [3,4]. First, a hazard assessment was performed. Therefore, studies describing the direct toxicity of the major components, i.e., the toxicity due to their direct effects, were summarized. For risk assessment, information on the smoking topography of young people using the shisha-pen (puffs per session, sessions per day, duration of use) is lacking. Therefore, possible risks were assessed by taking a pragmatic approach combining known topography for cigarettes [5] and e-cigarettes [6], using a 1-puff scenario (one-time exposure). The maximum concentrations of the major components in shisha-pen vapor that would reach the lower respiratory tract were calculated, as described previously [3,4].

In this study, the margin of exposure (MOE) approach was used as a procedure for risk assessment for components for which relevant human data were available. The MOE is the ratio of an appropriate toxicological Point of Departure (PoD) divided with the estimated human exposure; the smaller de ratio, the higher the risk. The MOE is evaluated given the necessary extrapolation steps involved whether clear conclusions can be drawn or if refinement is necessary. The latter is beyond the scope of the present paper. Basic calculations and a detailed description of the exposure and risk assessment steps have been previously described by Bos et al. [7]. In step 1, the exposure assessment is described, in step 2, the PoD, and in step 3, the risk on local effects is estimated.

## Results and discussion

A shisha-pen is an electronic inhaler that vaporises a liquid solution consisting mainly of humectants and flavors into an aerosol mist. Like e-cigarettes, shisha-pens simulate the act of tobacco smoking. In the present study, the contents and vapor of shisha-pens without nicotine with different flavors (apple, strawberry, grape) were analyzed.

The major components found in the liquid of shisha-pens were propylene glycol and glycerol (54%/46%). The manufacturer reports a minimum of 500 puffs to be taken from one shisha-pen (shisha-pen package). This was confirmed by our smoking machine analysis, in which up to 630 puffs were taken from one shisha-pen. GC analysis of shisha-pen puffs showed that the vapor in the shisha-pen was comprised of an average of 0.7 mg/puff of propylene glycol and 0.6 mg/puff of glycerol. In addition, the vapor contained a small amount of flavor and other trace components (<1%). No tri-ethyleneglycol, di-ethyleneglycol and nicotine were found. Furthermore, pyrolysis of shisha-pen vapor did not show presence of well-known tobacco smoke components, such as benzene or 1,3-butadiene.

Risk assessment was performed per major component found in shisha-pen vapor, propylene glycol and glycerol. The maximum concentration of propylene glycol and glycerol that would reach the lower respiratory tract after one puff was estimated, as described previously [3,4]. For the shisha-pen, the amount of puffs taken per unit time, the volume of vapor inhaled and the length of vaping sessions of the average shisha-pen user remain unknown. For this reason, smoking topography described for use of “normal” cigarettes [5] and e-cigarettes [8,9,6] were used. Calculations were made for a 1-puff scenario.

Propylene glycol is used in the food, cosmetic, pharmaceutical and plastic industries. It is also commonly used to create the artificial smoke or mist often seen in discotheques, theatre and television productions [10]. Glycerol is widely used in many industrial and consumer products, e.g. soaps/detergents, medicines, cosmetics, food, drinks, paints, resins and paper [10]. Both substances are “generally recognized as safe” (GRAS) for use as food additives [11]. The GRAS approval, however, does not apply to exposure to propylene glycol and glycerol through the shisha-pen. This is because in this scenario the substances are not ingested as in food, but inhaled, which results in exposure of the respiratory tract and lungs. For propylene glycol, it is known that repeated, short-term exposure of eyes, skin, nose, and mouth may cause irritation [12].

The concentrations that reached the airways and lungs after using a shisha-pen were compared to data from published toxicity studies [13-15]. Studies were selected based on resemblance of the exposure scenario to that of shisha-pen use. Differences between studies and the actual exposure to shisha-pen use, such as differences in duration of exposure and differences between animals and humans, were taken into account when only animal studies were available.

Hazard assessment of propylene glycol showed that there is no evidence that propylene glycol is carcinogenic to humans (The Health Council of the Netherlands [16]). Non-carcinogenic, local respiratory effects and systemic effects following propylene glycol exposure showed an increased number of goblet cells in the respiratory tract and nasal hemorrhaging observed when rats were exposed to 160 mg/m^3^ (the lowest concentration tested), 6 hours per day, 5 days per week for 13 weeks [14]. Effects such as nasal burning, stinging and throat irritation were attributed to exposure to propylene glycol as part of a pharmaceutical formulation inhaled by patients suffering from allergic rhinitis for 4 weeks. However, these effects were significantly less following a change in the content of propylene glycol in the formulation from 20% to 5% [17]. Furthermore, acute ocular and upper airway irritation was caused by short exposure to propylene glycol mist from artificial smoke generators in non-asthmatic human volunteers (n = 27) who were exposed in an aircraft simulator to propylene glycol mist for 1 minute. A few (4 out of 27) reacted with cough and slight airway obstruction [15]. Minor systemic effects were observed only in female rats which included body weight reduction and changes in leukocyte profile. These systemic effects on body weight and leukocyte profile have not been found consistently in other studies indicating that gender differences in susceptibility to propylene glycol’s adverse effects in the rat, but other studies do not provide additional evidence for this [17].

For risk assessment of propylene glycol the maximum alveolar concentrations in after one puff was estimated to be 430 to 603. The study of human volunteers (n = 27) exposed to propylene glycol for one minute at concentrations ranging from 176–851 mg/m^3^ showed upper airway irritation [15]. It is not clear if irreversible effects will occur after prolonged use but an animal study showed that repeated exposure (6 h per day; 5 days per week) for 90 days at 1000 and 2200 mg/m^3^ caused irreversible respiratory damage [14]. Limits for propylene glycol by actors exposed via theatrical fog has been set at 40 mg/m^3^ [18]. The estimated maximum alveolar concentration of propylene glycol in one puff exceeds this peak acceptable concentration. This analysis of the shisha-pen demonstrates that a risk of irritating effects on the respiratory tract epithelium due to propylene glycol exists. Details on risk assessment of propylene glycol (exposure assessment, PoD, and risk on local effects) is presented in Risk assessment propylene glycol; 1-puff scenario section (propylene glycol; 1-puff scenario). The MOE analysis is presented in Table 1.

**Table 1:** Summary MOE analysis, propylene glycol, 1-puff scenario, human study used as PoD

**Description**	**Selected study**
Critical endpoint	Acute irritation of the eyes and upper airways
	A few (4 out of 27) individuals reacted with additional cough and slight airway obstruction
Source	The Health Council of the Netherlands (2007) (Wieslander *et al.*, 2001)
Species	Healthy human subjects (27)
Exposure regimen	Propylene glycol in aviation emergency training. Exposure to artificial mist generator in short-term inhalation exposure (~1 min)
Concentrations tested (mg/m^3^)	0, 176-851
Duration of exposure	Average rating on 10 questions before and after 1 min propylene glycol exposure every 30 min for 4 hours
NOAEL (mg/m^3^)	-
If no NOAEL, then value for LOAEL	Min = 176
	Max = 851
	Mean = 309
*C*_*alv;max*_ (mg/m^3^)	430-603
Source of *C*_*alv;max*_ (mg/m^3^)	1 puff (50 mL [5], 70 mL [6])
MOE_1-puff shisha-pen/100% transfer rate_	***Min range = 0.3-0.4 (~2.5-3x higher than in human study)***
	***Max range = 1.4-2***
	***Mean range = 0.5-0.7 (~1.4-2x higher than in human study)***

### Risk assessment propylene glycol; 1-puff scenario

#### Step 1: Exposure assessment

For the exposure scenario, the same method as previously described [7] was utilized with a few adaptations. Puffing patterns (puff frequency, strength and duration) vary considerably among individuals who smoke electronic cigarettes or shisha-pens, but surveys indicate that individuals take an average of 120–175 puffs per day [8,9]. There is no data available on the duration of shisha-pen smoking sessions and therefore we can only assume that 1 puff has a volume of 50 mL as it is with cigarette smoke [5], or 70 mL as is found with e-cigarettes [6]. We must keep in mind that with the nicotine-free shisha-pen, the user will not adjust the volume to satisfy the nicotine craving, for this reason we used both volumes to obtain a range of exposure as an indication of the overall risk.

The average concentration per shisha-pen smoking session can be calculated by adapting the exposure scenario described previously for cigarette smoking [7] and dividing the amount in mg inhaled during a shisha-pen session (*D*_*1-puff shisha-pen*_) by 0.05 L^1^, or 0.07 L^2^.

- ^1^C_*alv;max*_ = 0.042 × *D*_*1-puff shisha-pen*_/0.05 = 0.85 × *D*_*1-puff shisha-pen*_ = mg/L
- ^2^C_*alv;max*_ = 0.042 × *D*_*1-puff shisha-pen*_/0.07 = 0.6 × *D*_*1-puff shisha-pen*_ = mg/L

GC analysis showed that the smoke in the shisha pen was comprised of an average of 0.71 mg/puff of propylene glycol:

- ^1^C_*alv;max*_ = 0.85 × *D*_*1-puff shisha-pen*_ = 0.85 × 0.71 mg = 0.603 mg/L = 603 mg/m^3^
- ^2^C_*alv;max*_ = 0.85 × *D*_*1-puff shisha-pen*_ = 0.6 × 0.71 mg = 0.43 mg/L = 430 mg/m^3^

The estimated inhaled concentration of propylene glycol per puff was 0.71 mg with a maximum alveolar concentration (*C*_*alv;max*_*)* of 430 to 603 mg/m^3^.

#### Step 2: Point of departure

One human study in which humans were exposed to an aerosol mist as part of an aviation emergency training was considered the best PoD for further risk assessment. Please refer to Table 1 for MOE calculation.

#### Step 3: Risk on local effects

The MOE for respiratory tract irritation was found to range from 0.3 to 2 (Table 1). Considering the MOE, a risk of effects on the respiratory tract epithelium due to propylene glycol exists. For evaluation of this MOE it needs to be taken into account that the lowest observed adverse effect level (LOAEL) was used as PoD instead of no observed adverse effect level (NOAEL).

Hazard assessment of glycerol showed no evidence for carcinogenic effects. Non-carcinogenic, local respiratory and systemic effects were reported as local irritant effects to the upper respiratory tract observed when rats were exposed to 662 mg/m^3^, 6 hours per day, 5 days per week for 13 weeks, with no toxic effects observed at 165 mg/m^3^ [19]. No systemic effects were reported in this study or in a study with rats exposed to concentrations of 1000, 1930 and 3910 mg/m^3^, 6 hours per day, 5 days per week for 14 days [19].

For risk assessment of glycerol, the maximum alveolar concentration of glycerol after one puff was estimated to be 348 to 495 mg/m^3^. Due to lack of relevant human inhalation studies with glycerol, no MOE was calculated. Nevertheless, two animal studies showed that continuous exposure (6 h per day; 5 days per week) for 14 and 90 days showed irritation to the upper respiratory tract at 662 and 1000 mg/m^3^, respectively [19]. Given the high inhaled concentration of glycerol in one puff, a risk of irritating effects on the respiratory tract epithelium due to glycerol exists with increased duration of shisha-pen exposure. Details on risk assessment of glycerol (exposure assessment, PoD, and risk on local effects) is presented in Risk assessment glycerol; 1-puff scenario section (glycerol; 1-puff scenario).

### Risk assessment glycerol; 1-puff scenario

#### Step 1: Exposure assessment

For the exposure scenario, the same method as previously described [7] was utilized with a few adaptations. Puffing patterns (puff frequency, strength and duration) vary considerably among individuals who smoke electronic cigarettes or shisha-pens, but surveys indicate that individuals take an average of 120–175 puffs per day [9,8]. There is no data available on the duration of shisha-pen smoking sessions and therefore we can only assume that 1 puff has a volume of 50 mL [5], or 70 mL [6]).

The average concentration per shisha-pen smoking session can be calculated by adapting the exposure scenario described previously for cigarette smoking [7] and dividing the amount in mg inhaled during a shisha-pen session (*D*_*1-puff shisha-pen*_) ) by 0.05 L^1^, or 0.07 L^2^.

- ^1^C_*alv;max*_ = 0.042 × *D*_*1-puff shisha-pen*_/0.05 = 0.85 × *D*_*1-puff shisha-pen*_ = mg/L
- ^2^C_*alv;max*_ = 0.042 × *D*_*1-puff shisha-pen*_/0.07 = 0.6 × *D*_*1-puff shisha-pen*_ = mg/L

GC analysis showed that the smoke in the shisha pen was comprised of an average of 0.582 mg/puff of glycerol

- ^1^C_*alv;max*_ = 0.85 × *D*_*1-puff shisha-pen*_ = 0.85 × 0.58 mg = 0.495 mg/L = 495 mg/m^3^
- ^2^C_*alv;max*_ = 0.6 × *D*_*1-puff shisha-pen*_ = 0.6 × 0.58 mg = 0.348 mg/L = 348 mg/m^3^

The estimated inhaled concentration of glycerol per puff was 0.58 mg with a maximum alveolar concentration (*C*_*alv;max*_*)* of 348 to 495 mg/m^3^.

#### Step 2: Point of departure

Two studies with continuous exposure were found. The first had an NOAEL of 165 mg/m^3^ and a LOAEL of 662 mg/m^3^ for local irritant effect to the respiratory tract in rats exposed 6 h per day, 5 days per week for 13 weeks (concentrations tested were 0, 33, 165 and 662 mg/m^3^) [19]. Another study showed an LOAEL of 1000 mg/m^3^ for local irritant effects of the upper respiratory tract in rats exposed 6 h per day, 5 days per week for 2 weeks (concentrations tested were 0, 1000, 1930 and 3910 mg/m^3^) [19]. It must be kept in mind that in the rat study, animals were exposed to glycerol for 6 h per day and that these data were compared with 1 puff of a shisha-pen.

#### Step 3: Risk on local effects

Because a relevant study with a similar exposure pattern as that of a shisha-pen could not be found, a reliable MOE could not be calculated. Nevertheless, the inhaled concentration of glycerol in one puff was estimated to be 348 to 495 mg/m^3^, in comparison to an NOAEL of 165 mg/m^3^, and an LOAEL of 1000 and 662 mg/m^3^ observed for local irritant effect in 2- and 13-week rat studies, respectively. Given the high inhaled concentration of glycerol in one puff, a risk of irritating effects on the respiratory tract epithelium due to glycerol exists with increased duration of exposure.

For the risk assessments performed for propylene glycol and glycerol present in the vapor of the shisha-pen, it is recognised that several assumptions have been made and that the risk assessment can be refined reconsidering these assumptions. Although such a refinement is beyond the scope of the present analysis, considering the low MOE, it remains to be seen if further refinement will alter the conclusion.

The current study is the first to present a chemical analysis and subsequent assessment of the risks of inhaling nicotine-free shisha-pen vapor, focusing on the major components propylene glycol and glycerol. For propylene glycol risk assessment could rely on a relevant human study, allowing for MOE analysis. Also, for glycerol animal data were available allowing for estimation of risks upon exposure. Some limitations include the lack of information on shisha-pen use; we can only assume that topography including puff volume is within the range of that of cigarettes and e-cigarettes. Furthermore, there were no human (propylene glycol) and animal (propylene glycol, glycerol) studies that mimicked the exposure scenario of shisha-pen smoking. Other factors to be taken into account when performing risk assessment include less-than-lifetime exposure, interspecies extrapolation (rat to humans), and inter-individual variability. Moreover, the current chemical risk assessment approach presents a single-component analysis and the combined effects of propylene glycol and glycerol need further investigation.

## Conclusions

In conclusion, upon use of a shisha-pen, consumers inhale propylene glycol and glycerol, resulting in exposure of the respiratory tract and alveolar space. This study shows that already after one puff of the shisha-pen the inhaled concentration is sufficiently high to potentially cause irritation of the airways. New products such as the nicotine-free shisha-pen and their potential popularity among elementary school children as was reported in the Netherlands, stress the need for detection of these products and assessment of their risks to inform regulatory actions aimed at limiting potential harm that may be caused to consumers and protecting young people to take up smoking. For the shisha-pen, further research needs to be directed to identification and assessment of the potency of the trace chemicals and possible other impurities that were found to be present (<1%) in its vapor. In addition, little information is known in regards to how young people use the shisha-pen (puffs per session, sessions per day, duration of use). Further research is needed to investigate how consumers, including young people use the shisha pen (puffs per session, sessions per day, duration of use). More information to fill these data gaps is needed to better assess the long-term risks of smoking shisha-pens.

## Abbreviations

GC: Gas chromatography
GC-MS: Gas chromatography–mass spectrometry
MOE: Margin of exposure
PoD: Point of Departure
LOAEL: Lowest observed adverse effect level
NOAEL: No observed adverse effect level
GRAS: Generally recognized as safe
